# Notes of Hospital Clinics

**Published:** 1897-08-14

**Authors:** 


### Notes of Hospital Clinics.

*[Reported for The Hospital, and corrected by the authors.]*

### CHARING CROSS HOSPITAL.

### A Clinical Lecture on "Some Points in the Treatment of Fractures."

By John Astley Bloxam, F.R.C.S., Senior Surgeon to the Hospital.

A clinical lecture was given at Charing Cross Hospital on July 8th, 1897, by Mr. Bloxam on "Some Points on the Treatment of Fractures."

A compound fracture may be direct or indirect, *i.e.*, it may be directly compound at the time, or may become compound through the tissues over the fracture sloughing. The greatest danger is sepsis, which is more likely to occur when the medullary canal is extensively opened. The first point is to carefully remove all clothing; then wash the skin over the neighbourhood of the wound with hot water and soap, and then with some antiseptic solution; then syringe out the wound with the antiseptic, and be particularly careful to wash the bone ends, and to remove all fragments of bone; and finally apply an antiseptic dressing. The structures around the broken ends are often severely injured, tendons partially divided, muscles torn and cellular spaces opened, &c.; so afterwards look out for spreading cellulitis with suppuration. When there is much bruising and consequently likely to be sloughs, the wound must be dressed more frequently, and the wound left open. If small it should be completely closed with collodium, and iodoform, and gauze. In all cases you have to consider the condition of the bone; you have to remove broken and detached portions—otherwise they may necrose. It is of no use to leave a fragment of bone uncovered by skin; this should be removed, and the protruding ends of the bone should be carefully and thoroughly cleansed before replacing them.

Before setting a fracture test the sensation of the parts below to see if there is any involvement of the nerves. Then examine the arteries below the fracture to see if they are intact. Then examine the veins. Venous bleeding is the most troublesome of any; and to deal with it it is often necessary to raise the limb. Be most careful how you apply bandages, taking great care that they do not constrict the limb at any part or press on any vein.

I remember a case of compound fracture of the lower third of the tibia and fibula which occurred some years ago. It was dressed, bandaged, and slung; but blood steadily oozed through the dressing, so the house surgeon was called up in the night, and another bandage was applied. But as it still bled other advice was taken. The bandages were removed, chloroform given, the wound opened and enlarged, and the bleeding vessels looked for, but none of any importance were found, and the man was replaced to the ward. However, oozing still continued, and when seen by myself the next morning the question of necessity of amputation was raised. The man was placed in the theatre again, and I noticed that after all the bandages had been removed there was no more bleeding; so, remembering this point, I de decided to attempt again to save the limb. So I put the fracture up, taking care to avoid all direct pressure, especially on the veins, and no further bleeding occurred.

The man, as you saw, was walking in the ward the other afternoon with a sound limb. This case shows the importance of observing the point above-mentioned —namely, the avoidance of putting pressure anywhere in the neighbourhood of the fracture, and not only in compound fractures, but in simple fractures does this rule apply. For in simple fractures there is, of course, no bleeding from constriction; but if you put pressure on the veins œdema is produced, and there is likely also to follow plugging of the veins. This may later on cause great trouble, such as an ulcer, or may delay union, and if union is delayed you may get fibrous—instead of bony—union. In all cases of fracture of the lower limb I look out for œdema, and directly I find such to exist I look to see if any pressure has been applied on the veins, either by the bandages or by the limb being improperly slung. By this point we see that indirectly a tight bandage may either cause delay, impair, or absolutely prevent union.

I cannot guard you sufficiently in the importance of care in applying a bandage in any fracture, and the avoidance of all pressure—certainly in the early stages of the injury. For I well remember some years ago a case of fractured patella, which, as we know, is a fracture involving the knee joint, and effusion into the joint is sometimes very great, leading to great separation of the fragments in consequence. Some people had recommended the wrapping of the limb in cotton wool, and the application of a bandage to prevent this effusion, and accordingly this had been done in this case. The knee was wrapped in cotton wool, and a bandage was applied over, and it was not thought at the time that the pressure was excessive—but it was, however, followed by gangrene, and the leg had finally to be amputated.

Fractures are set by making extension and by producing relaxation of the muscles at the time. Sometimes the extension is attempted permanently to be kept up by the aid of weights, &c.; in others, simply by the weight of the limb.

In the case of fracture of the upper part of the humerus. In setting the fracture the assistant stands behind the patient and fixes the scapula; then you grasp the elbow and extend. Then apply four splints around the limb, posteriorly from the fold of the axilla to the internal condyle, anteriorly from the tip of the acromion to the external condyle, externally from the spine of the scapula to the olecranon, and internally interrupted. This splint must be so applied as to avoid all pressure on the vessels, especially when the elbow is flexed, and extends from the fold of the axilla to the elbow. Then bandage round these four splints, and bend the elbow over the chest wall so as to bring the palm of the hand over the upper part of the sternum, and sling the hand, so letting the elbow hang loose and producing the extension.

In fractures of the middle of the humerus, place an angular splint on the inside reaching to the hand, because it is found that unless you fix the elbow-joint you are bound to get movement at the seat of fracture. Subsequently apply the other three splints as for fractures of the upper part of the humerus.

On going into the ward and explaining to the class the method of slinging fractures of the leg, Mr. Bloxam explained a point, viz., that we should always be careful that the straps by which the upper end of the posterior splint are slung from the cradle are not too long; otherwise the upper end of the heavy posterior splint hangs upon the thigh by means of the bandages, and these bandages being pulled tight press on the internal saphena vein, and so may produce œdema.

